# Promotion of Health

**Published:** 1920-02-14

**Authors:** 


**THE PUBLIC WELL-BEING—(*continued*).**

### Promotion of Health.

### English and International Progress.

INTERESTING and valuable progress in the organisation of infant and child welfare, not only in this country but throughout the world, was described at a recent meeting in the Mansion House. There really does seem great promise of a vast forward movement everywhere in the interests of the health of all peoples, and one of the questions on this week's agenda of the League of Nations' Council is a consideration of methods for undertaking the obligations regarding prevention and control of disease which the Covenant has placed upon the League.

Sir Arthur Stanley explained at the Mansion House meeting that a large number of excellent societies had sprung up for the betterment of the infant and the child, with the result that there had been considerable overlapping. The Central Council of Infant and Child Welfare sought to co-ordinate the good work that was being done. About a year ago there were twelve important societies engaged in this work. They all felt the need of coming together, and believed that by combining they would obtain better results. The twelve societies had combined under the ægis of the Red Cross, and had founded the Central Council of Infant and Child Welfare Work. It was not a part of that Council's operations to undertake any definite branch of the work itself —it would be a great mistake on their part to do anything to diminish the individuality of the different societies, but they desired to stand at the back of them, and do all they could to assist them to get money. They would only interfere when there was some danger of the societies overlapping. The Council had worked most harmoniously.

Dr. Marion Vaughan explained the practical features of the welfare centres, and pleaded for the continuance of the voluntary system in connection with their management. At these centres there should be no question of people's morals or manners, no question of the illegitimacy or religion of a child; the idea of a centre was that it should give advice to the mother for the benefit of her child. They liked to get into contact with young mothers. Their worst enemies were the grandmothers, who, of course, knew what was best for the child.

The world organisation was outlined by Sir Arthur Stanley, who declared that it would have been folly not to make use of the Red Cross organisation of the Allies during peace. They had founded the International League of Red Cross Societies, and they were a part of the League of Nations, but they had the additional point to their credit that they had been at work for months.

The object of the International League of Red Cross Societies was to gather together the best information that could be obtained on health and welfare work from every country in the world. The headquarters of the League were at Geneva, which would act as a clearing-house of ideas for every country belonging to the League. Departments for every form of health and relief work had been established at Geneva, including one devoted to infant and child welfare. The scheme was that in every country there should be a department corresponding with that of the headquarters at Geneva, and they had been asked to establish one in this country. The International League of Red Cross Societies had extended an invitation to the Central Council to become a corresponding member of their body at Geneva, and the Council had gladly accepted it. They were now in touch with child welfare organisations throughout the world. At the bottom of this movement lay the question of education, and they felt that by the course they had adopted they would be getting the best results. They would be having the advantage of the knowledge of all peoples of the world in dealing with the health and upbringing of our children at home.

It is probable that the League of Nations' Council will this week decide to appoint an Expert Committee to advise it on matters of international health.

